# Biogenic amines and their metabolites are differentially affected in the *Mecp2*-deficient mouse brain

**DOI:** 10.1186/1471-2202-12-47

**Published:** 2011-05-24

**Authors:** Nicolas Panayotis, Adeline Ghata, Laurent Villard, Jean-Christophe Roux

**Affiliations:** 1 INSERM UMR_S 910, Unité de Génétique Médicale et Génomique Fonctionnelle, Equipe de Neurogénétique Humaine, France; 2 Aix-Marseille Université, Faculté de Médecine de La Timone, Marseille, F-13385, France

## Abstract

**Background:**

Rett syndrome (RTT, MIM #312750) is a severe neurological disorder caused by mutations in the X-linked methyl-CpG binding protein 2 (*MECP2*) gene. Female patients are affected with an incidence of 1/15000 live births and develop normally from birth to 6-18 months of age before the onset of deficits in autonomic, cognitive, motor functions (stereotypic hand movements, impaired locomotion) and autistic features. Studies on *Mecp2 *mouse models, and specifically null mice, revealed morphological and functional alterations of neurons. Several functions that are regulated by bioaminergic nuclei or peripheral ganglia are impaired in the absence of *Mecp2*.

**Results:**

Using high performance liquid chromatography, combined with electrochemical detection (HPLC/EC) we found that *Mecp2*^-/y ^mice exhibit an alteration of DA metabolism in the ponto-bulbar region at 5 weeks followed by a more global alteration of monoamines when the disease progresses (8 weeks). Hypothalamic measurements suggest biphasic disturbances of norepinephrine and serotonin at pathology onset (5 weeks) that were found stabilized later on (8 weeks). Interestingly, the postnatal nigrostriatal dopaminergic deficit identified previously does not parallel the reduction of the other neurotransmitters investigated. Finally, dosage in cortical samples do not suggest modification in the monoaminergic content respectively at 5 and 8 weeks of age.

**Conclusions:**

We have identified that the level of catecholamines and serotonin is differentially affected in *Mecp2*^-/y ^brain areas in a time-dependent fashion.

## Background

**Figure 1:**
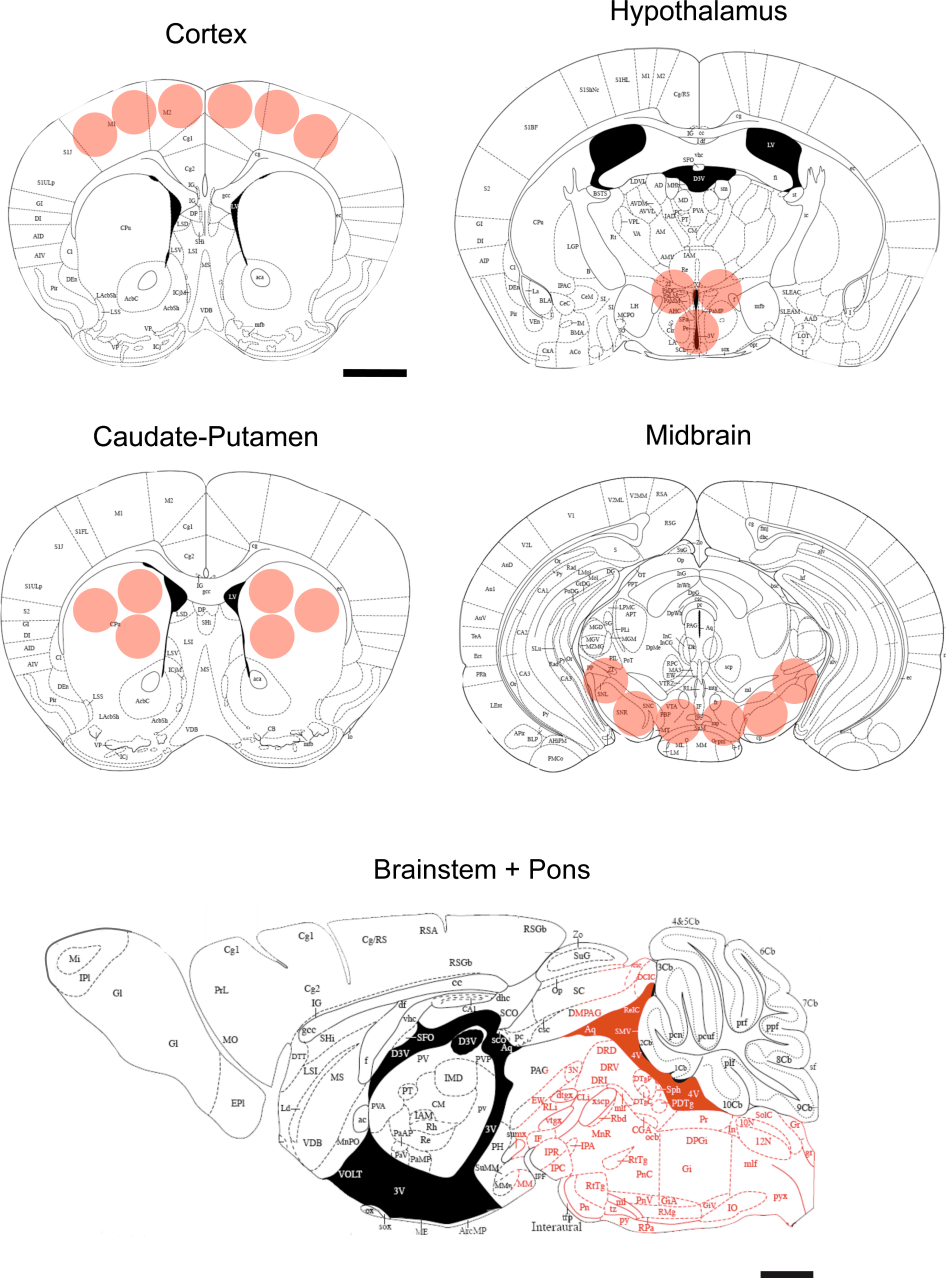
**Summary of sampled sites**. Cortical, Striatal (Caudate-Putamen), Hypothalamic, and Midbrain (Substantia Nigra + Ventral Tegmental Area) micropunched sites (respectively at +1.18, 0.98, -0.82, -3.08 from Bregma) and Brainstem + Pons area sampled mapped onto plates adapted from *The Mouse Brain in Stereotaxic Coordinates atlas *[39]. The pink circles/area mark sites were brain tissue was dissected out. Scale bars, 1 mm.

Rett syndrome (RTT, MIM #312750) is a severe neurological disorder caused by mutations in the X-linked methyl-CpG binding protein 2 (*MECP2*) gene [1]. Female patients are affected with an incidence of 1/15000 live births [2,3] and develop normally from birth to 6-18 months of age before the onset of deficits in autonomic, cognitive, motor functions and autistic features [4,5]. Several studies shed light on the involvement of catecholaminergic and serotonergic disturbances as a major contributor of the disease in RTT patients and *Mecp2 *mouse models. Neurochemical analyses of biogenic amines in *Mecp2*-null male (*Mecp2*^-/y^) were previously performed on whole brain homogenates showing that the concentration of dopamine (DA), norepinephrine (NE) and serotonin (5-HT) was reduced after birth [6,7]. Moreover, the absence of *Mecp2 *impairs several functions that are regulated by bioaminergic nuclei or peripheral ganglia where such neurochemical deficits occur [8-12]. However, these studies are discordant for several parameters. Here, we evaluated the level of DA, NE and 5-HT and their catabolites homovanillic Acid (HVA), dihydroxyphenylacetic acid (DOPAC) and 5-hydroxyindoleacetic acid (5-HIAA), using high performance liquid chromatography, combined with electrochemical detection (HPLC/EC). We performed our analyses on micropunched brain samples from the cortex, caudate-putamen, hypothalamus, midbrain (Substantia Nigra + Ventral Tegmental Area) and the hindbrain (Brainstem + Pons) (Figure 1) of *Mecp2*^-/y ^mice and their respective age-matched wild-type (WT) littermates from postnatal day 35 (P35, disease onset) and 55 (P55, severe phenotype).

## Results

### Neither catecholamines nor serotonin levels are affected in the cortex of *Mecp2*^-/y^

**Figure 2:**
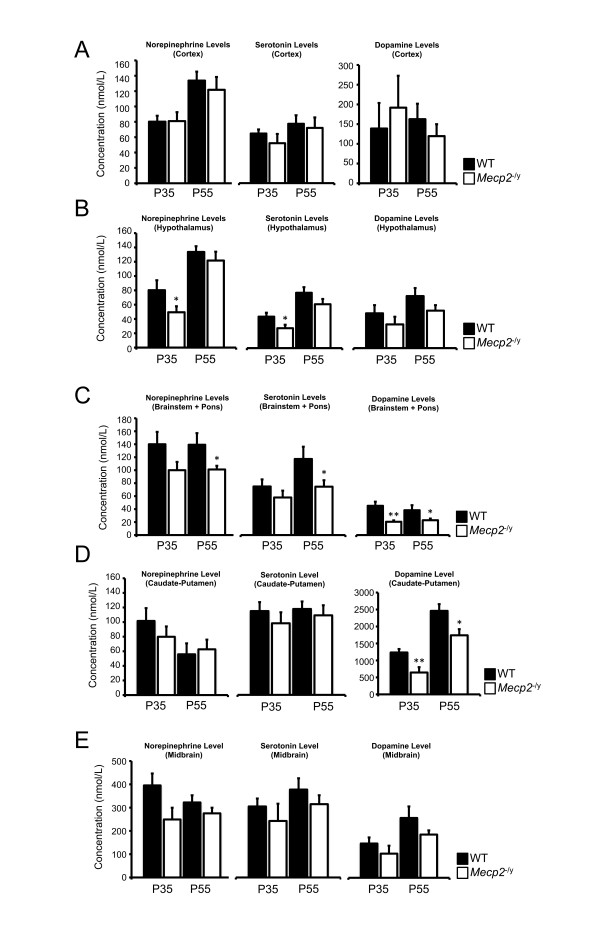
**Neurochemical analysis of catecholaminergic and serotonergic substrates**. Measure of norepinephrine, serotonin and dopamine content in the cortex (A, Cx), hypothalamus (B, Hyp), brainstem and pons (C, BP), caudate-putamen (D, CPu) and midbrain (E, Md) of *Mecp2*^-/y ^(white bars) and their WT littermates (black bars) at P35 (n = 6 *Mecp2*^-/y^, n = 10 WT for Cx, Hyp, BP and CPu - n = 6 *Mecp2*^-/y^, n = 9 WT for Md) and P55 (n = 9 *Mecp2*^-/y^, n = 8 WT for Cx, Hyp, BP, CPu and Md). Results are expressed as mean +/- S.E.M., (*p < 0.05, **p < 0.01).

**Figure 3:**
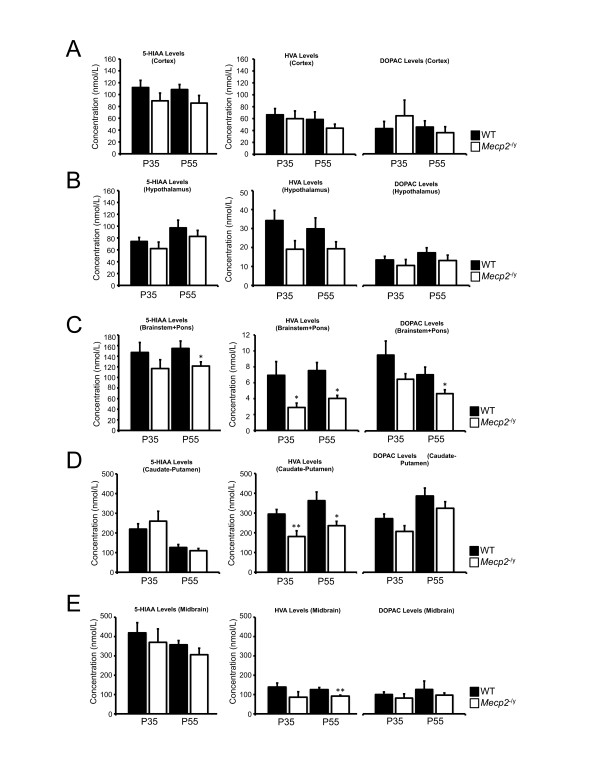
**Neurochemical analysis of dopaminergic and serotonergic catabolites**. Measure of homovanillic acid (HVA), dihydroxyphenylacetic acid (DOPAC) and 5-hydroxyindoleacetic acid (5-HIAA) content in the cortex (A, Cx), hypothalamus (B, Hyp), brainstem and pons (C, BP), caudate-putamen (D, CPu) and midbrain (E, Md) of *Mecp2*^-/y ^(white bars) and their WT littermates (black bars) at P35 (n = 6 *Mecp2*^-/y^, n = 10 WT for Cx, Hyp, BP and CPu - n = 6 *Mecp2*^-/y^, n = 9 WT for Md) and P55 (n = 9 *Mecp2*^-/y^, n = 8 WT for Cx, Hyp, BP, CPu and Md). Results are expressed as mean +/- S.E.M., (*p < 0.05, **p < 0.01).

Neurochemical analysis performed in the cortex showed no perturbation at P35 for DA, NE and 5-HT levels in *Mecp2*^-/y ^compared to their WT littermates (p > 0.05). No significant alterations were observed at P55 for DA, NE and 5-HT (p > 0.05). DA measurements in the cortex display great variability possibly masking differences between the two genotypes (Figure 2A). Dopaminergic catabolites HVA and DOPAC were unaffected at P35 and P55 (p > 0.05). The serotonin metabolite 5-HIAA is not different between *Mecp2*^-/y ^and WT at P35 and P55 (p > 0.05) (Figure 3A). Altogether, these results argue for a preserved biogenic amines metabolism in the cortex of *Mecp2*-null mice.

### The alteration of norepinephrine and serotonin levels in the hypothalamus of *Mecp2*^-/y ^is postnatal and biphasic

Next, we measured biogenic amines concentrations in the hypothalamus. It was previously reported that a targeted deletion of *Mecp2 *in *Single-minded 1 gene *(*Sim1)*-expressing neurons (located in several hypothalamic nuclei) results in a body of behavioural deficits ranging from feeding disturbances to stress [13]. Increased levels of corticosterone and leptin together with Brain-derived neurotrophic factor (Bdnf) deficits are possible contributors for this phenotype in this mouse. Since catecholamines play a role in hypothalamic nuclei neurotransmission and interplays with leptin and Bdnf [14-17] we wanted to assess deficits in bioamines and their degradation products. Deficits were observed at P35 for NE and 5-HT (p < 0.05). No alteration of DA levels was noticed at this age (p > 0.05). However, the NE and 5-HT decrease identified at P35 was no longer present at P55 and DA levels were normal at P55 (p > 0.05) (Figure 2B). All the tested catabolites were found to be normal in the hypothalamus of *Mecp2*^-/y ^compared to WT at P35 and P55 (Figure 3B).

### Bioaminergic disturbances are global and progressive in pontic and brainstem area of *Mecp2*^-/y^

DA, NE and 5-HT contents were evaluated by HPLC using pons and brainstem samples of *Mecp2*^-/y ^animals and WT animals at P35 and P55 (Figure 2C). DA is reduced both at P35 and at P55 (p < 0.05). NE levels are normal at P35 (p > 0.05) but are clearly reduced at P55 (p < 0.05). For serotonin, we found the same pattern of alteration, with no decrease at P35 (p > 0.05) but a decrease at P55 (p < 0.05). Altogether, our results suggest that *Mecp2*^-/y ^mice have lower levels of biogenic amines than their WT littermates.

We respectively assessed the level of HVA, DOPAC and 5-HIAA in these samples (Figure 3C). At P35, the only catabolite affected is HVA (p < 0.05) whereas DOPAC and 5-HIAA are normal (p > 0.05). At P55, the levels of HVA, DOPAC and 5-HIAA were all reduced in *Mecp2*^-/y ^compared to WT samples (p < 0.05).

### *Mecp2*^-/y ^nigrostriatal dopaminergic deficits do not parallel noradrenergic or serotonergic ones

We previously reported a postnatal reduction of DA and HVA but not DOPAC in the caudate-putamen of *Mecp2*^-/y ^at P35 and P55 [12] (illustrated on Figure 2D and Figure 3D). Here, we observed that at P35, neither NE nor 5-HT contents were affected (p > 0.05). This observation stands true for these neurotransmitters at P55 (p > 0.05). We measured the level of 5-HIAA and found no alteration at P35 (p > 0.05), and P55 (p > 0.05). These results indicate that the noradrenergic and serotonergic neurotransmitters are unaffected in the caudate-putamen of *Mecp2*^-/y ^compared to their WT littermates at all ages investigated (Figure 2D and Figure 3D).

Our previous work failed to detect changes in the level of DA and DOPAC in the *Mecp2*^-/y ^midbrain at both P35 and P55. However, we observed a clear reduction of HVA in these dopaminergic neurons at the most advanced age [12] (illustrated on Figure 2E and Figure 3E). We extended our analysis to NE, 5HT and 5-HIAA at both ages. Our results show that NE is not altered at P35 in *Mecp2*-deficient midbrain (p > 0.05). 5-HT levels are also preserved compared to WT level at the same age (p > 0.05). Samples obtained from symptomatic mice (P55) do no exhibit any modification of the substrate levels compared to age-matched WT (p > 0.05) (Figure 2E). 5-HIAA values are normal at P35 (p > 0.05) and at P55 (p > 0.05) in *Mecp2*^-/y ^samples compared to age-matched WT (Figure 3E).

## Discussion

*Mecp2*^-/y ^cortical neurons exhibit several alterations, ranging from reduced activity to abnormal dendritic spine morphology [18,19]. These defects are influenced by Bdnf dosage [20]. Bdnf plays a key role in neuronal survival, differentiation and synaptic plasticity [21]. Bdnf metabolism was shown to be involved in the development and function of catecholaminergic neurons, affected in *Mecp2*^-/y ^[22-25]. Recently, several studies identified NE reductions in the piriform cortex of *Mecp2*-null mouse at P35 [9] and in the prefrontal and motor cortex at both 3 (P21) and 8 (P56) weeks of age [11]. We were not able to reproduce these findings. One possible explanation for this divergence could be differences in the sampling procedure. We performed micropunches of the motor cortex. Different cortical territories receive monoaminergic inputs from different nuclei, some areas being less densely innervated than others [26]. Serotonergic dosage in *Mecp2*^-/y ^brains were also reported and discrepancies are existing. Isoda and collaborators did not to identify any modifications of 5-HT and its main catabolite 5-HIAA in the prefrontal cortex from P14 to P56 [10]. However, another laboratory argued for a reduction in the same structure of 5-HT only, at 3 and 8 weeks of age [11]. As Isoda and coauthors measured, we did not found significant reductions of 5-HT or 5-HIAA. Another group reported a 5-HT deficit in whole brain extracts in the same developmental window [7]. Unfortunately, samples used in this study came from pooled 6 to 8 weeks *Mecp2*^-/y ^animals, two ages that are know to be phenotypically different according to the postnatal development of the *Mecp2*^-/y ^mouse pathology [27]. These results thus combine 5-HT contents from both mildly and severely affected animals.

It is generally believed that catecholamines play a role in hypothalamic nuclei neurotransmission and that their release is modulated by several factors including Bdnf and leptin [17]. Here, we report a postnatal alteration of hypothalamic NE and 5-HT contents that follows a biphasic scheme. These biogenic amines were found significantly decreased at P35 in *Mecp2*^-/y ^animals whereas their levels are not significantly different from WT at P55. A recent study showed that specific deletion of *Mecp2 *in *Sim1*-positive neurons in the paraventricular nucleus (PVN) of the hypothalamus results in higher serum level of leptin [13]. Since leptin was proposed to lower NE-dependant oxytocin release in the PVN [17] and to reduce firing of serotonergic neurons [28] it was tempting to assess the level of bioamines in this brain area. Our results at P35 could reflect a leptin-dependant bioaminergic reduction. However, Fyffe and coauthors did not identify alterations at 6 weeks (P42) but noticed a significant increase 42 weeks. It is important to stress that *Mecp2*-deletion is not restricted to hypothalamic neurons in the model we used. Moreover, mice used in one study were of mixed 129/FVB genetic background [13] while we used C57Bl6 *Mecp2*^-/y ^mice [29].

In agreement with Taneja and co-workers [9], we found a reduction of DA and HVA in our hindbrain samples containing both the brainstem and the pons at P35. However, we did not found DOPAC reduction. Taneja et al. used a different *Mecp2*^-/y ^strain (i.e. generated by Jaenisch laboratory; [30]). It would be interesting to investigate possible difference in the activity/modulation of DA degradation enzymes in these two models. In the Bird mouse [29] no difference was found in monoamine oxydase (MAO-A and MAO-B) activity in the cortex [11]. It remains to be determined if MAO and/or catechol-O-methyl transferase (COMT) levels are impaired elsewhere. Interestingly, the caudal structures are the most affected with dopaminergic disturbances at P35 when the *Mecp2 *animals display a mild phenotype, and a global monoaminergic deficit at P55 affecting DA, NE, 5-HT and their degradation products. This is in good agreement with several studies arguing for a brainstem and pontic basis for the *Mecp2*^-/y ^phenotype and RTT symptoms in humans [31,32]. The observed increase in the serotonergic ratio (Table 1) at P55 in the pons and brainstem supports a possible alteration of 5-HT projections innervating this structure and modulating the autonomic functions altered in the absence of *Mecp2 *[33].

**Table 1:** Metabolite/5-HT and metabolite/DA ratios in the cortex, hypothalamus, caudate-putamen, midbrain and brainstem/pons of WT and *Mecp2*^-/^^y^.

Brain Area	Group	5HIAA/5HT ratio	(HVA + DOPAC)/DA ratio
**Cortex**	P35 Wt	1.68 (+/- 0.14)	1.71 (+/- 0.42)

	P35 Ko	1.88 (+/- 0.29)	1.33 (+/- 0.43)

	P55 Wt	1.50 (+/- 0.17)	0.75 (+/- 0.10)

	P55 Ko	1.47 (+/- 0.29)	0.77 (+/- 0.12)

**Hypothalamus**	P35 Wt	1.92 (+/- 0.26)	0.85 (+/- 0.06)

	P35 Ko	2.61 (+/- 0.37) *	0.94 (+/- 0.12)

	P55 Wt	1.37 (+/- 0.12)	0.73 (+/- 0.15)

	P55 Ko	1.54 (+/- 0.22)	0.62 (+/- 0.09)

**CaudatePutamen**	P35 Wt	1.91 (+/- 0.11)	0.47 (+/- 0.03)

	P35 Ko	2.64 (+/- 0.22) **	0.62 (+/- 0.05)

	P55 Wt	1.06 (+/- 0.13)	0.30 (+/- 0.02)

	P55 Ko	1.02 (+/- 0.08)	0.33 (+/- 0.02)

**Midbrain**	P35 Wt	1.51 (+/- 0.23)	1.66 (+/- 0.14)

	P35 Ko	1.79 (+/- 0.27)	1.64 (+/- 0.09)

	P55 Wt	1.06 (+/- 0.13)	1.07 (+/- 0.13)

	P55 Ko	1.02 (+/- 0.08)	0.99 (+/- 0.04)

**Brainstem + Pons**	P35 Wt	2.27 (+/- 0.39)	0.36 (+/- 0.03)

	P35 Ko	2.18 (+/- 0.31)	0.43 (+/- 0.09)

	P55 Wt	1.17 (+/- 0.06)	0.44 (+/- 0.06)

	P55 Ko	2.00 (+/- 0.51) *	0.39 (+/- 0.02)

Shown are mean values +/- S.E.M. for the ratios of dopaminergic metabolites HVA + DOPAC/DA and serotonergic metabolite 5-HIAA/5-HT in the cortex (Cx), the hypothalamus (Hyp), the caudate-putamen (CPu), midbrain (Md) and brainstem + pons (BP) of *Mecp2*^-/y ^and their WT littermates at P35 and P55. Dopamine turnover was unafected in the Cx, Hyp, CPu, Md and BP at both P35 and P55 in *Mecp2*^-/y ^compared to their age-matched WT littermates. Serotonergic turnover was not modified in Cx and Md at any age investigated. However, Hyp and CPu dosage revealed a significant increase of 5-HIAA/5-HT ratio at P35 but not P55. In turn, values from the BP indicate a clear increase in the serotonergic turnover in *Mecp2*^-/y ^when they exhibit the most severe phenotype (P55). (*p < 0.05, **p < 0.01).

*Mecp2*^-/y ^mice display a progressive and postnatal alteration in their motor behavior [11,34]. Interestingly, mice with a targeted deletion of *Mecp2 *in *Th*-expressing neurons show a deficit in motricity among other dysregulations [7]. We recently described in mice harboring a constitutive deletion of *Mecp2 *progressive alterations of the SNpc and the nigrostriatal dopaminergic pathway, a key component of the brain motor circuitry [12]. Moreover, some of the behaviors impaired were ameliorated by L-Dopa, arguing for a strong, although clearly non-unique, involvement of the DA disturbances in *Mecp2*^-/y ^motor phenotype. In complement to this previous study, we here pursued our neurochemical investigations for NE and 5-HT in the SNpc and its striatal target. NE, provided from the Locus Coeruleus, A1 and A2 (from brainstem) is implicated in several brain functions and its action on midbrain DA neurons was shown to modulate motivational behaviors [35,36]. The absence of NE and 5-HT disturbances in the midbrain and the caudate-putamen confirm previous findings [10,11].

The consequence of *Mecp2*-deficiency on the integrity of dopaminergic and serotonergic nuclei could be indexed on the HVA + DOPAC/DA and 5-HIAA/5-HT ratios respectively since a toxic alteration of dopaminergic and serotonergic systems induces counteradaptative processes that impact on this values [37,38]. The calculated ratios are reported in Table 1. Results suggest dopamine turnover was left unaffected in all the structures investigated at both P35 and P55 in *Mecp2*^-/y ^compared to their WT littermates. Conversely, hypothalamus and caudate-putamen dosage revealed a significant increase of 5-HIAA/5-HT ratio at P35 but not P55. Values from the pons and brainstem indicate a clear increase in the serotonergic turnover in *Mecp2*^-/y ^at P55. However, this index was not modified in the cortex and the midbrain at P35 and P55. Alteration of the serotonergic innervation lead to an increase in the turnover [37]. Interestingly, a reduction in 5-HT immunoreactive fibers was reported in the hippocampus of *Mecp2*^-/y ^animals [10]. It remains to be elucidated if such phenomenon contribute to serotonergic deficits described here.

## Conclusions

**Figure 4:**
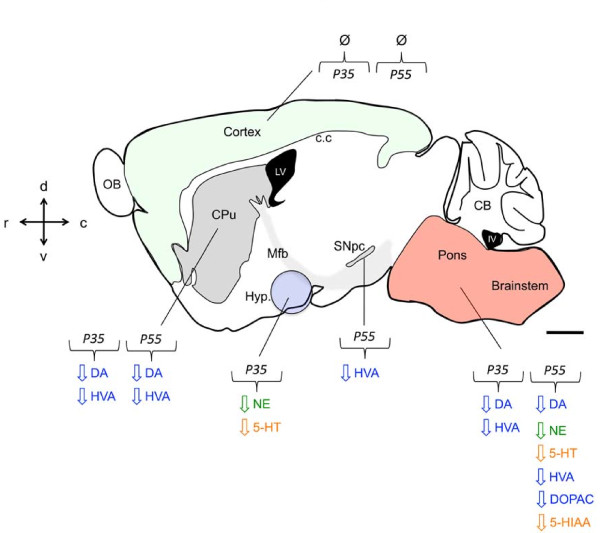
**Summary of catecholaminergic & serotonergic neurochemical measurements in the *Mecp2***^**-/y **^**brain**. In mouse, *Mecp2 *dysfunction leads to the gradual postnatal reduction of dopamine (DA), norepinephrine (NE), serotonin (5-HT) and their principal degradation product dihydroxyphenylacetic acid (DOPAC), homovanillic acid (HVA) and 5-hydroxyindolacetic acid (5-HIAA) in several neuronal populations. These monoaminergic nuclei-related perturbation result in characterized phenotypes that constitute some of the key features of RTT pathology in humans. Sagittal mouse brain drawing adapted from *The Mouse Brain in Stereotaxic Coordinates atlas *[39]. Area fill in green depict the cortical mantle. In gray the substantia nigra pars compacta (SNpc) irradiating the caudate-putamen (CPu) through the medial forebrain bundle (mfb). Hypothalamic (Hyp) region is symbolized in purple and the pons + brainstem in pink. OB (olfactory bulb), c.c (corpus callosum), CB (cerebellum), LV (lateral ventricle), IV (4^th ^ventricle). Scale bar, 1 mm.

Our results indicate that the level of catecholamines and serotonin is differentially affected in *Mecp2*^-/y ^brain areas in a time-dependent fashion (Figure 4). Considering behavioral and physiological function altered in the context of *Mecp2*-deficiency and the positive impact of drugs targeting this system, monoamine neurochemical dosage could help identifying therapeutics resetting central bioaminergic deficits in different brain areas of the *Mecp2*-deficient mouse.

## Methods

### Animals

Experiments were performed on the B6.129P2(c)-*Mecp2*^tm1-1Bird ^mouse model for RTT [29]. The mice were obtained from the Jackson Laboratories and maintained on a C57Bl/6 background. The experimental procedures were carried out in keeping with the European guidelines for the care and use of laboratory animals (Council Directive 86/609/EEC). Both pre-symptomatic and symptomatic mice were analyzed at different developmental stages. A total number of 15 *Mecp2*^-/y ^and 18 WT male mice were used in the study. The *Mecp2 *^-/y ^(null male) and WT (wild-type male) mice were studied at postnatal days 35 (P35; n = 10 WT; n = 6 *Mecp2*^-^^/y^) and 55 (P55; n = 8 WT; n = 9 *Mecp2*^-/y^) for neurochemical analysis. *Mecp2*-deficient mice were compared to their respective WT littermates of the same gender. Breeding and genotyping were performed as previously described [23].

### Tissue sampling

P35 (n = 10 WT; n = 6 *Mecp2*^-/y^) and P55 (n = 8 WT; n = 9 *Mecp2*^-/y^) mice were killed by cervical dislocation, and their brains were dissected out within the first 2 min post-mortem. The motor cortex, Caudate-Putamen, Midbrain area (Substantia Nigra + Ventral Tegmental Area) were microdissected using a punching needle (0.5 mm ∅) and kept at -80°C until biochemical analysis. The region containing both the Pons and the Brainstem was dissected under a binocular microscope (Figure 1). Briefly, brain area dissection was performed on cryostat brain sections with the help of a 5× magnifying lens, following their stereotaxic coordinates [39,40].

### Chemicals

Norepinephrine, DHBA, dopamine, DOPAC, HVA, serotonin (5-hydroxytryptamine, 5-HT), 5-hydroxyindole-3-acetic acid (5-HIAA), 1-octanesulfonic acid (OSA), triethylamine and ethylene-diamine-tetra-acetic acid (EDTA) disodium salt were purchased from Sigma, sodium dihydrogen phosphate and citric acid from Merck and methanol from Prolabo. Ultrapure water was obtained with a Milli-Q system (Millipore, Bedford, MA, USA). Standard solutions of each monoamine or metabolite were stored at -20°C at 1 mmol/L as aliquots.

### HPLC

The HPLC system was composed of a Hitachi L-7000 series equipped with a degasser, a L-7100 pump, an L-7200 autosampler thermostated at 10°C and a Decade Intro electrochemical detector fitted with a 3 mm glass carbon working electrode, an Ag/AgCl reference electrode and a 25 μm spacer (Antec, Leyden, The Netherlands). Separations were performed using a 250 mm × 4.6 mm i.d. C18 5 μm Beckman Ultrasphere column equipped with two Phenomenex C18 filters in a security guard system. The mobile phase was pumped at a microflow rate of 0.8 mL/min and composed of 0.7 mol/L sodium phosphate, 0.1 mmol/L EDTA, 1.1 mmol/L OSA, 3.1 mmol/L triethylamine, 14% methanol, pH adjusted to 3.12 with 1 mmol/L citric acid, it was filtered with 0.45 μm cellulose acetate membranes before use. Elutes were detected at an oxidation potential of 700 mV versus reference electrode. The column and the detection cell were housed within the Faraday cage of the electrochemical detector that was set to 25.5°C. The day of the analysis, 35 μL samples were placed in the autosampler and kept at +10°C before injection. The injection volume was 30 μL. The retention times were 7 min, 11 min, 13.5 min, 16.5 min, 21 min, 32 min and 42 min for NE, DHBA, DOPAC, DA, 5-HIAA, HVA, and 5-HT, respectively.

### Statistical analysis

We evaluated whether our data distribution fitted with a Gaussian representation using a K-S Kolmogorov-Smirnov Normality test. If valid, data were statistically analyzed using unpaired Student's *t *test. If not valid, we used an adapted non-parametric Mann-Whitney test to compare genotypes. The results are reported as mean +/- standard error of the mean (S.E.M). A p-value < 0.05 was considered to be statistically significant.

## Authors' contributions

LV and JCR conceived and designed the experiment. NP and AG performed the experiments. NP and JCR analyzed the data. NP and JCR wrote the paper. All authors read and approved the final manuscript.
